# Overgrowth-intellectual disability disorders: progress in biology, patient advocacy and innovative therapies

**DOI:** 10.1242/dmm.052300

**Published:** 2025-05-12

**Authors:** Cooper Atterton, Isabella Trew, Jessica M. Cale, May T. Aung-Htut, Kerry Grens, Jill Kiernan, Christal G. Delagrammatikas, Michael Piper

**Affiliations:** ^1^ The School of Biomedical Sciences, The University of Queensland, Brisbane, QLD 4072, Australia; ^2^ Personalised Medicine Centre, Health Futures Institute, Murdoch University, Murdoch, WA 6150, Australia; ^3^ Centre for Neuromuscular and Neurological Disorders, Perron Institute for Neurological and Translational Science, Nedlands, WA 6009, Australia; ^4^ TBRS Community, Stanfordville, NY 12581, USA; ^5^ Malan Syndrome Foundation, Old Bridge, NJ 08857, USA; ^6^ The Queensland Brain Institute, The University of Queensland, Brisbane, QLD 4072, Australia

**Keywords:** Overgrowth, Intellectual disability, Malan syndrome, Tatton-Brown-Rahman syndrome, Sotos syndrome, Cerebral cortex

## Abstract

Overgrowth-intellectual disability (OGID) syndromes encompass a group of rare neurodevelopmental disorders that frequently share common clinical presentations. Although the genetic causes of many OGID syndromes are now known, we lack a clear mechanistic understanding of how such variants disrupt developmental processes and ultimately culminate in overgrowth and neurological symptoms. Patient advocacy groups, such as the Overgrowth Syndromes Alliance (OSA), are mobilising patients, families, clinicians and researchers to work together towards a deeper understanding of the clinical needs of patients with OGID, as well as to understand the fundamental biology of the relevant genes, with the goal of developing treatments. In this Review, we summarise three OGID syndromes encompassed by the OSA, namely Sotos syndrome, Malan syndrome and Tatton-Brown-Rahman syndrome. We discuss similarities and differences in the biology behind each disorder and explore future approaches that could potentially provide a way to ameliorate some of the unmet clinical needs of patients with OGID.

## Teaser

**Summary:** Overgrowth-intellectual disability disorders share common clinical phenotypes, yet have different aetiology. A comprehensive understanding of these differences, commonalities and treatment possibilities is crucial for ensuring informed patient-centred care.

## Introduction

Overgrowth-intellectual disability (OGID) syndromes are a family of rare genetic neurodevelopmental disorders, which often have significant overlaps in their clinical profiles (Grens et al., 2024). Broadly, overgrowth is defined as a head circumference and/or height greater than two standard deviations above the mean (Manor and Lalani, 2020), and intellectual disability (ID) ranging from mild cognitive impairment to severe intellectual disability, often concomitant with a co-diagnosis of autism spectrum disorder (ASD) (Lee et al., 2024; Khachadourian et al., 2023). Although the genetic cause of some OGID disorders has been clear for decades, in many other instances this has not been the case, and the advent of genetic testing has allowed for a clearer understanding of the biological drivers of OGID syndromes. As a result, the discovery and classification of the disorders continues to grow, such that there are at least 13 recognised OGID disorders (Table 1), as well as additional segmental overgrowth disorders (a phenotype of overgrowth that is confined to one or a few regions of the body) (Grens et al., 2024; Manor and Lalani, 2020). Genetic variants affecting a range of different proteins – including epigenetic regulators and chromatin modifiers, transcription factors and cell signalling kinases – have been identified as causative of different OGID syndromes (Grens et al., 2024). However, the way in which variants of relevant OGID genes lead to altered developmental trajectories and subsequent patient symptoms remains unclear in most cases.

**Table 1.** **O** **vergrowth-intellectual disability syndromes included in the Overgrowth Syndromes Alliance**

Disorder name	Causative gene	References
Overgrowth-intellectual disability syndromes		
Beck-Fahrner syndrome (OMIM #618798)	*TET3* (OMIM #613555)	Fahrner (2023)
CHD8-related neurodevelopmental disorder (OMIM #615032)	*CHD8* (OMIM #610528)	Mitchel et al. (2022)
Cohen-Gibson syndrome (OMIM #617561)	*EED* (OMIM #605984)	Sequerra Amram Cohen and Gibson (2019)
Imagawa-Matsumoto syndrome (OMIM #618786)	*SUZ12* (OMIM #606245)	Imagawa et al. (2023)
NF1-SUZ12 deletion syndrome	*NF1* (OMIM #162200) and *SUZ12* (OMIM #606245)	Kehrer-Sawatzki et al. (2021)
Kosaki syndrome (OMIM #616592)	*PDGFRB* (OMIM #173410)	Takenouchi et al. (2019)
Luscan-Lumish syndrome (OMIM #616831)	*SETD2* (OMIM #612778)	Pappas and Rabin (2022)
Malan syndrome (OMIM #614753)	*NFIX* (OMIM #164005)	Priolo (2024)
Simpson-Golabi-Behmel syndrome (OMIM #312870)	*GPC3* (OMIM #300037)	Nisbet et al. (2024)
Smith-Kingsmore syndrome (OMIM #616638)	*MTOR* (OMIM #601231)	Besterman et al. (2021)
Sotos syndrome (OMIM #117550)	*NSD1* (OMIM #606681)	Tatton-Brown et al. (2022)
Tatton-Brown-Rahman syndrome (OMIM #615879)	*DNMT3A* (OMIM #602769)	Ostrowski and Tatton-Brown (2022)
Weaver syndrome (OMIM #277590)	*EZH2* (OMIM #601573)	Ocansey and Tatton-Brown (2024)
Segmental overgrowth syndromes		
CLOVES syndrome* (OMIM #612918)	*PIK3CA* (OMIM #171834)	Mirzaa et al. (2023)
Macrocephaly-capillary malformation* (OMIM #602501)		
Proteus syndrome* (OMIM #176920)	*AKT1* (OMIM #164730)	Biesecker and Sapp (2023)
Beckwith-Wiedemann syndrome* (OMIM #130650)	*CDKN1C* (OMIM #600856) *KCNQ1OT1* (OMIM #604115) *ICR1* (OMIM #616186)	Shuman et al. (2023)

*Not represented by the Overgrowth Syndromes Alliance at the time of publication. CLOVES, congenital lipomatous overgrowth, vascular malformations, epidermal nevi, and skeletal/spinal anomalies; OMIM, Online Mendelian Inheritance in Man.

The identification of causative genes has spurred the formation of many patient advocacy groups, whose goals encompass research, improvements to clinical care, family support and the development of treatments for these disorders. One such example is the Overgrowth Syndromes Alliance (OSA), a recently formed collective supporting patients and families with overgrowth disorders including Sotos syndrome (SS), Tatton-Brown-Rahman syndrome (TBRS) and Malan syndrome (MALNS) (Box 1). These disorders, despite arising from different genetic diagnoses, exhibit significant overlaps in clinical presentation, as well as educational, emotional and social needs of the affected patients and their families (Table 2). In this Review, we offer a patient advocacy perspective, highlighting the importance of community-based organisations in connecting patients, families, researchers and clinicians to ultimately improve the lives of those affected with OGID syndromes. We also discuss the prospect of shared biological pathways contributing to common clinical presentations in patients with SS, MALNS and TBRS, summarising what is known about how the genes involved mediate brain development and function, thereby potentially revealing common threads linking these overgrowth disorders. Finally, we discuss antisense oligonucleotides (ASOs) as a future treatment option for these syndromes, as these offer a promising pathway to the clinic and are being used with some success to treat other neuromuscular disorders, such as spinal muscular atrophy (Mercuri et al., 2018; Chiriboga et al., 2016; Finkel et al., 2016).

**Box 1. The Overgrowth Syndromes Alliance and its role in patient-focused outcomes**

The Overgrowth Syndromes Alliance (OSA) is a collaborative initiative formed in 2023 by the Tatton Brown Rahman Syndrome Community and Malan Syndrome Foundation to address the fragmented research and advocacy efforts surrounding overgrowth-intellectual disability (OGID) syndromes. Encompassing 13 distinct genetic conditions that share significant clinical, phenotypic and molecular characteristics, the OSA aims to unite the overgrowth community around shared patient priorities and advance comparative research to improve the quality of life and clinical outcomes for individuals living with these conditions.

As a key first step, the alliance conducted a community survey to better understand the priorities and concerns of patients and their families. Guided by these patient priorities, the OSA is taking strategic action to address critical clinical and research gaps. A cornerstone of this effort is the planned establishment of multidisciplinary centres dedicated to OGID syndromes, which will provide comprehensive, specialised care while facilitating robust data collection. The OSA is advancing educational initiatives through a monthly speaker series featuring overgrowth syndrome experts and planning for in-person scientific meetings to foster knowledge sharing and collaborative research. In addition, OSA patient advocacy organisations have worked collaboratively to raise awareness about the prevalence of seizures and epilepsy across multiple overgrowth syndromes, successfully publishing a paper on this topic within its first year of formation (Grens et al., 2024). Throughout these efforts, OSA maintains active communication with diverse stakeholders, including patients, advocacy groups, researchers, clinicians and the broader rare disease community, ensuring that findings and progress are widely shared.

The OSA has built a substantial international research network comprising more than 120 professionals interested in overgrowth conditions. Of the 13 OGID syndromes (Grens et al., 2024), currently five (Tatton-Brown-Rahman syndrome, Sotos syndrome, Malan syndrome, Smith-Kingsmore syndrome and Beck-Fahrner syndrome) have established patient advocacy organisations, highlighting the progress made and the opportunity for further development of support and research networks.

**Table 2.** Common features conserved between Sotos syndrome, Malan syndrome and Tatton-Brown-Rahman syndrome, and their reported prevalence

Clinical feature	Prevalence in SS (Tatton-Brown et al., 2022)	Prevalence in MALNS (Priolo, 2024)	Prevalence in TBRS (Ostrowski and Tatton-Brown, 2022)
Characteristic facial appearance	• ≥90%	• 100%	• Not reported – only readily clinically evident in adolescence
Intellectual/learning disability	• ≥90%	• 100%	• 100%
Overgrowth*	• ≥90%	• Macrocephaly: 100% • Height: 56%	• >80% • Macrocephaly: ∼50% • Height: ∼70%
Overweight/underweight**^‡^**	• Birth weight normal (Tatton-Brown et al., 2022) • Overweight uncommon (Waggoner et al., 2005)	• Low BMI: 38% • Birth weight above average: 90%	• Overweight: ∼65%
Behavioural findings, i.e. autism spectrum disorder	• 15-89%	• 86% • 67% showed hypersensitivity to noise	• ∼50% • 44% met formal criteria for autism
Advanced bone age	• 75-80% of prepubertal children	• 76%	• Not reported
Cardiac anomalies	• 20%	• 30% (Huynh et al., 2024)	• Aortic root dilation: ∼3% (Totten et al., 2024)
Cranial MRI/CT abnormalities	• 15-89%	• 25-50% • Chiari malformation: 38% (Macchiaiolo et al., 2022)	• Chiari malformation: <10%
Joint hyperlaxity/pes planus	• >20%	• Pes planus: 69%	• Joint hypermobility: ∼75%
Neonatal complications	• Jaundice: ∼65% • Hypotonia: ∼75% • Poor feeding: ∼70%	• Hypotonia: 65%	• Hypotonia: ∼55%
Scoliosis/kyphoscoliosis	• ∼30%	• 75%	• ∼30%
Seizures/epilepsy	• ∼25% to ∼50%	• 46-63% (Huynh et al., 2024)	• ∼20%
Tumours/malignancies	• ∼3%	• ∼2%	• ∼5% • ∼150 haematologic malignancies/million (>250-fold increased risk) (Ferris et al., 2022)
Cryptorchidism	• ≥2% to <15%	• 13%	• ∼20%

*Overgrowth is defined as height and/or head circumference ≥2 s.d. above the mean for age and sex. ^‡^Overweight (underweight) is defined as weight ≥2 s.d. above (≤2 s.d. below) the mean for age and sex. BMI, body mass index; CT, computed tomography; MALNS, Malan syndrome; MRI, magnetic resonance imaging; SS, Sotos syndrome; TBRS, Tatton-Brown-Rahman syndrome.

## Sotos syndrome

### Clinical presentation

First identified in 1964 (Sotos et al., 1964), SS [Online Mendelian Inheritance in Man (OMIM) #117550] is the most common OGID syndrome, occurring in ∼1 in 14,000 live births (Tatton-Brown et al., 2022). Patients with SS have a distinctive appearance characterised by a broad and prominent forehead, dolichocephalic head shape, downslanting palpebral fissures, long/narrow face and chin, and overgrowth (Tatton-Brown et al., 2022). Learning disabilities are also often present, manifesting as early developmental delay and ID ranging from mild to severe (Tatton-Brown et al., 2005). Other features frequently seen in patients with SS are summarised in Table 2 (Tatton-Brown et al., 2022). Genetic analyses in 2002 revealed the cause of SS to be loss-of-function variants of nuclear receptor-binding SET-domain protein 1 (*NSD1*) (Kurotaki et al., 2002). Early studies confirmed that *NSD1* contains 23 exons (Kurotaki et al., 2001) and is located on chromosome 5 (5q35.3) (Jaju et al., 2001). In a cohort of 239 *NSD1*-positive individuals, 83% were identified as having intragenic variants resulting in heterozygosity for *NSD1* and 10% had 5q35 microdeletions (Tatton-Brown et al., 2005). Genetic testing is also recommended in all diagnoses, as there are several conditions that present similarly to SS but are distinct entities. This is most evident from several cases of suspected SS being diagnosed clinically, but then diagnosed with a different OGID syndrome following genetic testing (Ostrowski and Tatton-Brown, 2022). These include TBRS, Cohen-Gibson syndrome (Sequerra Amram Cohen and Gibson, 2019), Beckwith-Wiedemann syndrome (Shuman et al., 2023), Simpson-Golabi-Behmel syndrome (Klein et al., 2024) and Weaver syndrome (Ocansey and Tatton-Brown, 2024).

### Causative gene biology

*NSD1* (OMIM #606681) encodes a nuclear protein consisting of multiple domains, including two nuclear receptor interaction domains (NID^−L^ and NID^+L^) that direct its interactions *in vivo* (Kurotaki et al., 2001). Human *NSD1* produces three isoforms (Tauchmann and Schwaller, 2021), and displays high structural and sequence conservation in comparison to mouse *Nsd1* (Tauchmann and Schwaller, 2021; Kurotaki et al., 2001). NSD1 is a lysine methyltransferase (Tauchmann and Schwaller, 2021; Morishita and di Luccio, 2011) that mediates the mono- and di-methylation of lysine (K) 36 on histone H3 (H3K36me2) (Morishita and di Luccio, 2011), with these marks having key roles in processes including chromatin accessibility and RNA polymerase II occupancy and progression (Lucio-Eterovic et al., 2010). NSD1 also plays key roles in the methylation of other histone [i.e. H4K20 (Berdasco et al., 2009)] and non-histone [i.e. reversible methylation of K218/K221 of the p65 subunit of NF-κB *in vitro* (Lu et al., 2010)] substrates, highlighting its importance across development (Tauchmann and Schwaller, 2021).

**Fig. 1.**
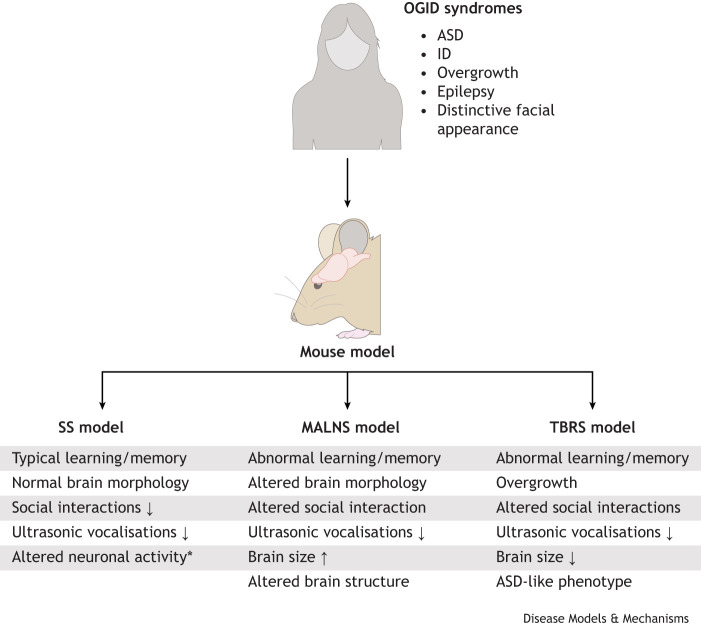
**Conserved clinical phenotypes map into mouse models of each disorder.** A common clinical diagnostic core typifies the presentation of many overgrowth-intellectual disability (OGID) syndromes, and this is similarly conserved for Sotos syndrome (SS), Malan syndrome (MALNS) and Tatton-Brown-Rahman syndrome (TBRS). Models of these disorders have shown that many of these neural-based symptoms are recapitulated in heterozygous models of each disorder, suggesting a conserved function of the causative genes across development in mice and humans. *Finding shown from homozygous ablation, not heterozygous ablation. ASD, autism spectrum disorder; ID, intellectual disability.

Few studies have been published attempting to model SS; however, these models have provided some useful insights. One of the first SS modelling studies did not recapitulate the overgrowth phenotype observed in patients with SS, as neither the body weight nor head size (either macrocephaly or megalencephaly) were increased (Oishi et al., 2020). The authors showed no deficits in learning using the active place avoidance test (a hippocampal-dependent learning task), but did show defects in socialisation behaviours using the three-chamber social interaction test, as well as a measure of the abnormal mother-pup dyad through ultrasonic vocalisations. These tests have both been reliably used in the past to assess autism-like behaviours (Bronzuoli et al., 2018; Premoli et al., 2021), a common feature of patients with SS (Tatton-Brown et al., 2022). However, it is important to note that the use of a hippocampal-dependent learning task, such as active place avoidance, may miss other learning abnormalities that are hippocampal independent. As such, it is important for future studies on these models of OGID syndromes to include both hippocampal-dependent and -independent tasks, such as novel object recognition (Denninger et al., 2018), to ensure that all possible phenotypes are investigated and elucidated. Another model of SS used various cortical-specific Cre drivers to ablate *Nsd1* activity from the developing cortex instead (Zheng et al., 2023). The authors then assessed conditional knockout mice and found that ablation of *Nsd1* from the entire forebrain or from postmitotic pyramidal neurons resulted in abnormally developed brains, altered behavioural responses and disrupted transcriptional and methylation landscapes (Fig. 1). These knockout models highlighted similarities in psychosocial, cognitive and motor phenotypes that may present in the SS cohort (de Boer et al., 2006).

## Malan syndrome

### Clinical presentation

First reported in 2010 (Malan et al., 2010), MALNS (OMIM #614753) was previously referred to as Sotos syndrome 2 prior to its identification as a distinct entity, given the high degree of similarity in clinical presentation. The incidence of this disorder is estimated at 2.6 in 100,000 live births (Lopez-Rivera et al., 2020). Patients with MALNS present with a characteristic appearance, comprising prenatal and postnatal overgrowth, macrocephaly, advanced bone age and/or skeletal abnormalities (such as scoliosis), as well as distinctive facial features (including a long and triangular face, high anterior hairline, downslanted palpebral fissures and prominent chin). These features also typically present alongside intellectual and learning disabilities (Priolo, 2024) (summarised in Table 2). The causative allele of MALNS is nuclear factor one X (*NFIX*) (Malan et al., 2010), a transcription factor belonging to a larger family of NFI genes that are essential for typical development of numerous organ systems (Campbell et al., 2008). *NFIX* contains 11 exons (Grunder et al., 2003) and is located on chromosome 19 (19p13.3) (Seisenberger et al., 1993). MALNS has been described to result broadly from a heterozygous pathogenic variant in *NFIX* (∼75% of affected individuals) or a heterozygous deletion of 19p13.2 (∼25% of affected individuals), which encompasses *NFIX* and other adjacent genes (Priolo, 2024).

### Causative gene biology

*NFIX* (OMIM #164005) belongs to a multi-gene family that encodes site-specific CCAAT-binding transcription factors capable of initiating transcription of both vertebral and viral genes (Campbell et al., 2008). *NFIX* interacts with double-stranded DNA in either a hetero- or homo-dimer via sequence recognition binding of the palindromic sequence TTGGC(N5)GCCAA (Gronostajski, 2000), exhibiting high affinity and allowing either repression or activation of gene transcription depending on the cellular context (Harris et al., 2015). Human *NFIX* has at least six splice variants (Apweiler et al., 2023), with the canonical human *NFIX* isoform exhibiting ∼90% sequence homology with the mouse *Nfix* gene (Apweiler et al., 2023). Outside of their ‘canonical’ roles as transcriptional activators or repressors in various signalling pathways crucial for cell cycle processes, proliferation and differentiation (Zhou et al., 2018; Heng et al., 2015; Heng et al., 2014; Martynoga et al., 2013), the NFI family has also been demonstrated to have regulatory roles in epigenetic modifications (Fane et al., 2017). Specifically, the proline-rich C-terminal domain (also known as the transactivation domain) (Gronostajski, 2000) of NFIs has been shown to interact with both histone H1 (Dusserre and Mermod, 1992) and H3 (Alevizopoulos et al., 1995). This is thought to occur through chromatin interactions, resulting in increased chromatin accessibility and gene expression (Pjanic et al., 2013), as well as through interactions with epigenetic complexes (such as the nucleosome remodelling enzyme complex NURF), which drive increases in other epigenetic marks (such as H3K4me3 and H3K36me3) (Pjanic et al., 2011). Regardless, the NFI family, and indeed NFIX, play crucial distinct roles in the developmental regulation of several organ systems and contexts (Harris et al., 2015).

The first models of *NFIX* deficiency (Campbell et al., 2008; Driller et al., 2007) were generated prior to the advent of MALNS as a diagnosis, but still provide interesting mechanistic insights into the neural developmental phenotypes that drive a suspected diagnosis of MALNS. Previous studies into *Nfix*-deficient mice have revealed many conserved phenotypes with the human syndrome presentation (Fig. 1). An early study on *Nfix* biology revealed increased brain wet weight (Campbell et al., 2008), suggestive of possible brain overgrowth. This was further substantiated by another study on *Nfix* deficiency confirming increased cortical thickness, concurrent with increased neuronal numbers in the cortex, as well as an enlarged brain when assessed using diffusion tensor magnetic resonance imaging (Oishi et al., 2019). These mice displayed abnormal connectivity and axonal integrity, which translated into behavioural abnormalities resembling those seen in patients, such as autistic-like phenotypes, deficits in socialisation and cognitive defects (Fig. 1) (Oishi et al., 2019).

## Tatton-Brown-Rahman syndrome

### Clinical presentation

First reported in 2014 (Tatton-Brown et al., 2014), TBRS (OMIM #615879) is another OGID syndrome that appears clinically like the group of ‘Sotos-like’ syndromes. The incidence of this disorder is estimated at 2.9 in 100,000 live births, and there have been at least 90 cases reported in the literature as of 2022 (Ostrowski and Tatton-Brown, 2022). TBRS, being a relatively newly identified disorder, has no consensus for clinical diagnostic features to suggest a diagnosis; however, the presence of certain clinical findings (Table 2) – including generalised overgrowth, developmental delay and ID, dysmorphic facial features, musculoskeletal and/or neurological abnormalities and behavioural issues – warrants genetic testing (Ostrowski and Tatton-Brown, 2022). In 2014, the cause of TBRS was identified to be heterozygous loss-of-function variants of the DNA methyltransferase *DNMT3A* (Tatton-Brown et al., 2014); however, a large proportion of *DNMT3A* variants have been identified as missense variants (Christian et al., 2020). *DNMT3A* contains 26 exons (Weisenberger et al., 2002) and is located on chromosome 2 (2p23.3) (Xie et al., 1999). Heterozygosity for *DNMT3A* pathogenic variants has been identified as the primary cause of TBRS, but there have been case reports of larger deletions of 2p23 encompassing *DNMT3A*, which causes a TBRS-like phenotype (Okamoto et al., 2016).

### Causative gene biology

*DNTM3A* (OMIM #602769) belongs to a family of DNA methyltransferases (Lyko, 2018). Mammalian DNMT enzymes contain an N-terminal regulatory domain and a C-terminal catalytic domain (Lyko, 2018), as well as two domains important for chromatin interactions, namely the proline-tryptophan-tryptophan-proline (PWWP) and ATRX-DNMT3A-DNMT3L (ADD) domains (Du et al., 2015; Tajima et al., 2022). *DNMT3A* acts as a *de novo* DNA methyltransferase, as it can bind to unmethylated or hemi-methylated DNA and catalyse the addition of a methyl group to 5-cytosine (5mC) (Zhang et al., 2018). *DNMT3A* produces two isoforms (DNMT3A1 and DNMT3A2) that exhibit differential patterns of localisation during development (Manzo et al., 2017). DNMT3A1 is 130 kDa in size, while DNMT3A2 is 100 kDa as it lacks the N-terminal domain found in DNMT3A1 (Chen et al., 2002). Through its role as a DNA methyltransferase, DNMT3A is poised to have several crucial roles outside of 5mC catalysis – these include transcriptional repression/activation, transposon silencing and cell fate specification (Lyko, 2018). As such, DNMT3A plays an important role in the development of an organism, as, in its absence, postnatal impairment and cell-type-specific defects ensue (Okano et al., 1999; Jones and Liang, 2009). Excellent reviews summarise the structural composition of DNMT3A and its isoforms in greater detail than presented here (Tajima et al., 2022; Lyko, 2018).

TBRS, despite being newly characterised, has one of the most complete and accurate mouse models of an OGID syndrome to date. Multiple studies have converged on common findings (Christian et al., 2020; Beard et al., 2023), with mouse models that recapitulate not only the overgrowth and physiological parameters, but also many of the behavioural differences commonly seen in patients with TBRS (Fig. 1). One of the first studies attempting to model TBRS, via heterozygous deletion of the *Dnmt3a* gene (Christian et al., 2020), demonstrated overgrowth (obesity onset in adulthood, increased tibia length and subtle differences in cranial structures), altered behavioural responses, and abnormal methylation and transcriptional profiles, which likely correlate with those seen in patients with TBRS (Chen et al., 2021). In collaboration with the same laboratory, another group further expanded upon these findings (Smith et al., 2021), suggesting a common function of R878H mutations (R882H in humans) and deletion of exon 19 and a similar (albeit weaker) effect of the P900L mutation (P904L in humans) (Beard et al., 2023). These studies are suggestive of and provide evidence for the hypothesis that the type of variant affects the severity of presentation in TBRS, as is the case for many other disorders and cancers (Ostrowski and Tatton-Brown, 2022).

## Clinical and mechanistic overlap between OGID syndromes

OGID syndromes, including those under the OSA banner, share many clinical phenotypes (Table 2). This raises the question: are these shared ‘overgrowth-intellectual disability’ symptoms reflective of commonly dysregulated pathways and mechanisms during development? Animal models have provided key insights into this – below, we review research that has begun to reveal how NSD1, NFIX and DNMT3A shape development, with a focus on how they mediate development of the nervous system. We also look forward and discuss how some of the emerging technologies in the biomedical arena may enable a deeper understanding of the mechanisms controlled by these factors, from which we hope to define dysregulated shared and divergent pathways that ultimately converge on clinical symptoms.

### Impacts on neural development

Overgrowth of the brain, also known as megalencephaly, in *Nfix*-deficient mice is driven by prolonged neural stem cell self-renewal and is potentially the underlying cause of macrocephaly in MALNS (Oishi et al., 2019); but is this conserved for other OGID syndromes, such as SS and TBRS? Although there are identified roles for both *NSD1* and *DNMT3A* in regulating stem cell biology (Fang et al., 2021; Challen et al., 2011), there is currently little evidence to suggest that brain overgrowth is dysregulated in the same way. Previous studies on mice heterozygous for *Nsd1* showed no brain overgrowth and no major dysmorphic features (Oishi et al., 2020). However, there are several possibilities that were not investigated in previous studies that could be driving brain overgrowth in other OGID syndromes. Abnormalities in neuronal migration (Buchsbaum and Cappello, 2019) and dendritogenesis (Hokkanen et al., 2012) have both been demonstrated to contribute to megalencephaly. Although the apparent cause of megalencephaly in *Nfix*-deficient mice has been identified, it does not preclude other processes from being dysregulated and contributing to the phenotype (Fig. 1). Investigation of these other related processes in other OGID mouse models could reveal a common phenotype that underlies the macrocephalic phenotype in humans – indeed, evidence suggests that *Nsd1* ablation in postmitotic neurons causes increased spine density and differences in excitatory post-synaptic potential (Zheng et al., 2023), and other studies highlight possible roles for *Nfix* in regulating dendritogenesis (Harris et al., 2018). These possibilities could be assessed through multi-omics sequencing (Trevino et al., 2021), allowing simultaneous profiling of both the transcriptional landscape and chromatin architecture at the single-cell level, as has been performed in part previously (Harris et al., 2018; Zheng et al., 2023). Comparative analyses can then be performed between models to understand whether there are commonalities that exist in the dysregulated biology – indeed, this seems likely as *NFIX* has been shown to interact with histone H3 previously (Alevizopoulos et al., 1995), which is the main regulatory target for *NSD1* activity (Tauchmann and Schwaller, 2021), suggesting a possible conserved pathway through which *NFIX* and *NSD1* are acting in the human condition.

### Epigenetic regulation of gene expression

**Fig. 2.**
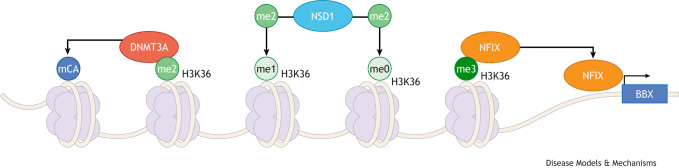
**An overview of the interactions between DNMT3A, NSD1 and NFIX.** The causative genes for SS, MALNS and TBRS have been identified as *NSD1*, *NFIX* and *DNMT3A*, respectively; however, there is a high degree of biological overlap. NSD1 deposits the dimethyl mark (H3K36me2, green) on lysine 36 (K36) of histone H3, using either the unmethylated (H3K36me0) or monomethylated (H3K36me1) forms (light green) as a substrate. DNMT3A requires H3K36me2 as a substrate prior to its activity as a DNA methyltransferase – once H3K36me2 bound, DNMT3A can deposit non-CG methyl marks (mCA; blue). Additionally, NFIX has been shown to interact with H3K36me3 (dark green), a mark deposited by SETD2 (the encoding gene is causative of another OGID syndrome, Luscan-Lumish syndrome) (Pappas and Rabin, 2022) upon substrate recognition of NSD1-deposited H3K36me2. NFIX recognition of H3K36me3 determines where NFIX should bind to drive transcription [such as near the promoter of downstream transcription factors, like Bobby Sox (BBX) (Dixon et al., 2013)], highlighting interaction between *NSD1*, *NFIX* and *DNMT3A* in several different contexts.

Many studies have been performed in both human tissues (Chen et al., 2021; Levy et al., 2022) and mouse models (Christian et al., 2020; Challen et al., 2011) to better characterise the episignature associated with DNA methylation catalysed by DNMT3A. However, few studies have characterised how DNMT3A interacts with other regulatory molecules to define the methylation landscape. A recent study (Hamagami et al., 2023) investigating *Nsd1* function revealed that NSD1 defines where non-CG methylation can and cannot be deposited, and that alterations in DNA methylation and gene expression resultant from *Nsd1* disruption overlap with those seen in a mouse model of TBRS (Fig. 2). These findings highlight an important biological overlap between *Nsd1* and *Dnmt3a* in mice and suggest that similar mechanisms are occurring in the human conditions. Indeed, it has been known for over a decade that histone modifications and DNA methylation patterns interact and overlap in human genetics and diseases (Miller and Grant, 2013). Additionally, a recent study (Velasco et al., 2021) using DNA methylation (DNAme) profiling performed comparative methylome analyses on a number of samples from various patients with OGID, including patients with SS, TBRS and Luscan-Lumish syndrome (Table 1), as well as a number of genetically related disorders. The authors found overlapping patterns of methylation across different disorders. It is perhaps no surprise then that until the advent of genetic testing, many of these now-distinct entities were all thought to be variants of one syndrome; hence, clinical genetic testing is now an absolute requirement for accurate differentiation (Tatton-Brown et al., 2022; Priolo, 2024; Ostrowski and Tatton-Brown, 2022).

### Complementary methods for OGID syndrome modelling

Given the overlapping functions of *DNMT3A*, *NFIX* and *NSD1*, as described below (see Fig. 2), a natural extension of the current research landscape should be the development and characterisation of trans-heterozygote mice, mimicking the likely overlap suspected to be occurring in the human condition. Trans-heterozygote models of *DNMT3A* and *NSD1* would be useful to better understand both TBRS and SS, and the development of other related trans-heterozygote models would also be warranted [i.e. *NSD1* and SET domain containing 2 (*SETD2*), which cause SS and Luscan-Lumish syndrome, respectively] (Pappas and Rabin, 2022). Development and characterisation of these trans-heterozygote models is essential to understanding the full breadth of the dysregulation that is occurring in OGID syndromes and will help to better inform targeted treatment possibilities moving forward.

Techniques that can robustly interrogate how *Nsd1* heterozygosity shapes both the histone modification and chromatin landscapes, such as CUT&RUN (Skene and Henikoff, 2017), will prove invaluable in complementing this endeavour. CUT&RUN allows for profiling of histone modifications, and has been used to investigate *NSD1* function previously (Drosos et al., 2022), although not in the context of SS. The use of this technique would reveal whether marks outside of H3K36me2 are also dysregulated in *Nsd1* heterozygous mice or patients with SS. Importantly, CUT&RUN has been used for neuronal-specific interrogation previously (Pollina et al., 2023), suggesting its suitability for investigation of possibly disturbed neuronal pathways in the absence of one *Nsd1* allele. Identification of targets that are altered through techniques such as CUT&RUN, when coupled with more canonical sequencing methods such as single-cell RNA sequencing (Wamsley et al., 2024), will provide insight into possible dysregulated pathways and hopefully provide better avenues for treatments.

In addition, there are a plethora of other techniques that can be used alongside these to help improve our depth of understanding of OGID syndromes. It is important for researchers in these spaces to be well versed in both the older ‘classics’ of neuroscience research and simultaneously at the forefront of new technologies. The use of techniques such as electrophysiology on *ex vivo* cultured brain slices (Hess et al., 2023) and *in vivo* recordings of electrical activity (Patel et al., 2020) is informative at both the single-cell level and more broadly when modelling OGID syndromes. This is perhaps most relevant when considering the incidence of epilepsy and seizures, which have been noted across many OGID syndromes (Grens et al., 2024). Understanding how dysregulation of these genes contributes to seizures will allow better understanding of the underlying biology, and also better management and treatment of persistent seizures.

The use of cell-culture and organoid modelling techniques is also invaluable. Organoids allow the translation of traditional two-dimensional *in vitro* cell cultures, which could only shed light on some mechanistic processes, into a three-dimensional version of the organ, recapitulating many different facets of biology (Luo et al., 2016). Rapid advances in this field have meant that it is now possible to create ‘mini-brains” that recapitulate brain developmental stages, and it is now possible to induce mutations using CRISPR in cells (e.g. induced pluripotent stem cells, or otherwise) (Huber et al., 2023) and grow into organoids, or even produce three-dimensional organoid systems from patient cells (Yang et al., 2018). Coupling these methods with technologies such as multi-electrode arrays (which allow for non-invasive and high-speed recording of extracellular electric field potential) (Huang et al., 2022) would not only allow elucidation of the underlying epileptiform activity in these patient-derived organoids (Nieto-Estevez and Hsieh, 2020) but also how brain activity may be altered in patients with OGID. Altogether, the future of OGID research is an incredibly exciting place to be, and there are many advances waiting to be made.

## Current management strategies for OGID syndrome patients

OGID disorders, including those encompassed by the OSA, share many clinical features. As such, management of SS, MALNS and TBRS involves a common range of interventions, which are outlined briefly below. For a more comprehensive review of all management guidelines, excellent reviews exist (Tatton-Brown et al., 2022; Priolo, 2024; Ostrowski and Tatton-Brown, 2022).

### Neurobehavioral phenotypes and interventions

The first indications of a neurobehavioral abnormality in patients with OGID disorders come in the form of hypotonia in infancy and delayed acquisition of motor skills in toddlers. These findings have been consistently noted across SS (Lesinskiene et al., 2024; Tatton-Brown et al., 2022), MALNS (Priolo, 2024; Huynh et al., 2024; Macchiaiolo et al., 2022) and TBRS (Tatton-Brown et al., 2018; Ostrowski and Tatton-Brown, 2022). These findings tend to resolve in most cases, although they can persist. However, early treatment of hypotonia is crucial, requiring involvement of an interprofessional team, including physical, occupational and speech therapists (Huynh et al., 2024; Makker et al., 2024). Additionally, school-based interventions can be useful in resolving motor delays should they not resolve in early development (Bryce, 2021). ID, ranging from severe disability to normal cognitive functions, has been reported in patients with SS (de Boer et al., 2006; Lesinskiene et al., 2024; Foster et al., 2019), MALNS (Macchiaiolo et al., 2022; Alfieri et al., 2022) and TBRS (Lane et al., 2020; Tenorio et al., 2020). Continued educational support, behavioural interventions and vocational training have all been shown to assist people with overgrowth and ID (Lee et al., 2024) and should be recommended for all patients.

ASD is often co-morbid with ID, and 21.7% of patients in the general population have ASD (Khachadourian et al., 2023). This co-morbidity is likely significantly elevated in the OGID cohort, as previous studies have estimated that ∼83% of SS (Lane et al., 2019), ∼31% of MALNS (Priolo et al., 2018) and ∼44% of TBRS (Lane et al., 2020) patients have ASD. Similarly to ID, behavioural therapy and environmental therapy have been suggested to improve the daily functioning and lives of people with ASD (Mughal et al., 2024). A less prominent feature is sensory processing deficits, which have been observed in patients with SS, MALNS and TBRS (Smith et al., 2023; Mulder et al., 2020). These sensory differences (which are often co-morbid with ASD) (Marco et al., 2011) hamper typical responses to a person's environment and can impact involvement in daily activities, as well as daily functioning (Mulder et al., 2020). Treatment can involve therapy, as well as identification and mitigation of sources of sensory distress (i.e. different lights for photosensitivity, using tinted glasses to improve emotional regulation) (Camarata et al., 2020; Ludlow et al., 2020).

### Other clinical manifestations and their treatments

Sleep disturbances have been reported in many patients with OGID, with reports of∼69% of patients with SS (Lesinskiene et al., 2024) and ∼71% of patients with MALNS (Huynh et al., 2024) experiencing some form of disturbance, and case reports suggesting that some patients with TBRS present with sleep apnoea (Balci et al., 2020). Non-apnoea disturbances commonly include early bed and rise times and repetitive motions following sleep onset – disturbances that result in sleep patterns differing from other patients with ID (Stafford et al., 2021). Behavioural programs (such as sleep hygiene and behavioural reinforcement), as well as pharmacological interventions, can be useful in patients with ID who have sleep disturbances (Harper et al., 2023). For patients with sleep apnoea, the use of a continuous positive airway pressure (CPAP) machine can be highly beneficial (McDaid et al., 2009); however, compliance with CPAP usage can pose difficulties for patients with OGID syndromes, when considering ID, sensory and behavioural challenges (Luijks et al., 2017).

Seizures and epilepsy are relatively common in patients with OGID, with estimates between ∼25% and ∼50% in SS (Tatton-Brown et al., 2022; Lourdes et al., 2023; Grens et al., 2024), between ∼46% and ∼63% in MALNS (partially owing to deletions encompassing *NFIX* and other genes such as *CACNA1A*) (Priolo, 2024; Bellucco et al., 2019; Grens et al., 2024; Huynh et al., 2024), and at ∼20% in TBRS (Ostrowski and Tatton-Brown, 2022; Tatton-Brown et al., 2018; Grens et al., 2024). Treatment is typical, involving the use of anti-epileptic medications (Stafstrom and Carmant, 2015). Musculoskeletal abnormalities are common, with patients presenting with scoliosis in ∼30% of SS and TBRS cases, and ∼75% of MALNS cases (Table 2). Meanwhile, pectus excavatum is present in <15% of patients with SS (Tatton-Brown et al., 2022) and ∼63% of patients with MALNS (Priolo, 2024), while appearing to be absent from the TBRS cohort (Ostrowski and Tatton-Brown, 2022; Tenorio et al., 2020, 2018). Treatment for scoliosis involves observation, bracing or surgery depending on the degree of curvature present (Janicki and Alman, 2007). Standard treatments for pectus excavatum typically include pain management (if indicated), use of a vacuum bell and/or surgery, depending on the severity (Abid et al., 2017).

Finally, cancer surveillance is not typically recommended in SS, MALNS and TBRS, which all have malignancy estimates of less than ∼5% (Table 2). However, where indicated, cancer surveillance should be followed up in routine checkups, or if other underlying conditions are met (Lapunzina, 2005; Villani et al., 2017).

## Antisense oligonucleotides, a possible bright future for OGID treatment?

As discussed, the current standard of care for SS, TBRS and MALNS is limited to management of symptoms, with a lack of treatments capable of targeting the underlying pathologies (Grens et al., 2024). The development of a therapy that modulates the root cause of, or a disease-associated pathway in, these disorders would dramatically improve quality of life for patients and their families. This could involve the use of gene therapy to target the causative genes *NSD1*, *DNMT3A* and *NFIX* directly, or others involved in shared pathways. The most common examples of gene therapy include the use of viral vectors, CRISPR-based editing and ASOs.

Viral vectors operate through the introduction of a synthetic ‘healthy’ copy of the affected gene delivered within a modified virus (Bulcha et al., 2021). As the synthetic gene is located on a plasmid that does not integrate into the genome and therefore may not be subject to endogenous regulatory mechanisms, expression at higher levels in cells or at times in which it does not naturally occur could be deleterious. CRISPR-based therapies operate through the direct editing of a patient's genome by modulating endogenous repair pathways to knock out the gene, alter its sequence to render it dysfunctional or correct disease-causing variants (Uddin et al., 2020). The requirement for a protospacer adjacent motif sequence (which determines the site of CRISPR editing), and heterogeneity of disease-causing variants, make the considerations for CRISPR-based therapies complex. Furthermore, viral vectors are commonly used to deliver the CRISPR machinery into cells, which incur high immunotoxicity (Uddin et al., 2020).

In comparison to these and other gene therapy methods, ASOs allow for the highly specific modulation of gene expression – which is perhaps a crucial consideration for dose-sensitive genes, as is the case in SS, TBRS and MALNS, in which overexpression may be deleterious. ASOs are single-stranded nucleic acid analogues, typically 15-30 bases in length, that target endogenous RNA to alter gene expression in a range of pathologies. Half of the 13 US Food and Drug Administration (FDA)-approved ASO drugs are utilised to treat nervous system disorders, with many more involved in current pre-clinical and clinical trials. These include ASOs developed to treat neurodevelopmental disorders such as Angelman syndrome (NCT04259281 and NCT05127226), Dravet syndrome (NCT04442295 and NCT04740476), and SCN2A developmental and epileptic encephalopathy (NCT05737784). In addition to treatments for these rare neurological disorders, their use as personalised therapies for nano- and ultra-rare disorders (e.g. *n*=1) continues to gain momentum after the 2018 approval of Milasen, a variant-specific ASO designed for a patient with severe Batten's disease (Kim et al., 2019). Subsequent patient-specific ASOs have followed this precedent, such as Atipeksen for the treatment of an individual with the rare neurological syndrome ataxia-telangiectasia (Kim et al., 2023) and Valeriasen for an individual with epilepsy arising from a variant of *KCNT1* encoding a sodium-activated potassium channel (Burbano et al., 2022).

### ASO mechanisms

**Fig. 3.**
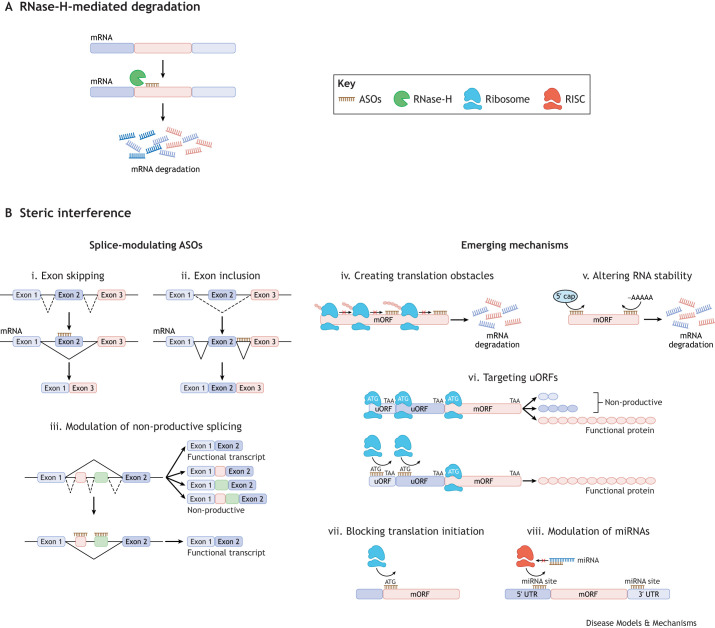
**Antisense oligonucleotide mechanisms of action.** (A,B) Overview of antisense oligonucleotide (ASO) mechanisms categorised by whether they operate through the activation of endogenous RNase-H degradation pathways to decrease target gene expression (A) or steric interference of RNA processes to modulate target gene expression (B). (A) Binding of an ASO to target mRNA creates an RNA:DNA duplex, which is recognised and selectively cleaved by the endogenous nuclease RNase-H. (B) The most established steric interference approach is the modulation of endogenous pre-mRNA splicing processes, which may include skipping (i) or inclusion (ii) of an exon, or modulation of non-productive splicing (iii). Other steric interference mechanisms currently emerging include the creation of translation obstacles (iv) that trigger ribosomal stalling and the no-go decay pathway, altering RNA stability (v) by disrupting the formation of the 5′ cap or polyadenylation (-AAAAA) sites, targeting upstream open reading frames (uORFs) (vi) to increase ribosome affinity to the main open reading frame (mORF), blocking ribosome binding or the formation of the initiation complex at the mORF (vii), and modulating microRNA (miRNA) molecules (viii) by blocking their binding site on target RNA or inhibiting entry to the RNA-induced silencing complex (RISC). UTR, untranslated region.

ASOs precisely target endogenous RNA and, therefore, do not affect gene expression in locations or stages of development in which it is not naturally expressed. This is an important advantage for OGID syndromes, whereby development is impacted. Many additional attributes make ASOs promising candidates for therapeutic development, including their remarkable specificity to not only a single gene target but a particular sequence 15-30 bases long. This is due to their single-stranded nucleic acid structure that facilitates binding to a complementary target sequence through Watson-Crick base pairing, at which they act to alter gene expression through a variety of mechanisms. Such mechanisms are classified into two groups: RNase-H-mediated degradation (Fig. 3A) and steric blocking (Fig. 3B), depending on whether they act by cleavage or interference of the target nucleic acid sequence (Crooke et al., 2021).

ASOs such as Tofersen [used to treat a subpopulation of patients with amyotrophic lateral sclerosis (Miller et al., 2022, 2020)] and GTX-102 [an ASO currently undergoing Phase I/II clinical trials for Angelman syndrome (Dindot et al., 2023)] utilise RNase-H-mediated degradation mechanisms. However, over half of the approved ASO drugs sterically interfere with endogenous splicing processes, resulting in the inclusion or exclusion of particular exons, or manipulation of non-productive splicing (Fig. 3B, i-iii). Examples include Nusinersen, an ASO approved for the treatment of spinal muscular atrophy (Mercuri et al., 2018; Chiriboga et al., 2016; Finkel et al., 2016); Eteplirsen, the first of four ASOs that restore low levels of dystrophin production in patients with Duchenne muscular dystrophy (Mendell et al., 2013; Cirak et al., 2011); and Milasen, a variant-specific ASO approved for the treatment of a patient with severe Batten's disease (Kim et al., 2019).

Although the only steric blockade mechanism approved to date is modulation of splicing (Fig. 3B, i-iii), there are a plethora of other mechanisms that are now approaching the clinic (Fig. 3B, iv-viii). These include creating translation obstacles that trigger the no-go decay (NGD) pathway (Fig. 3B, iv) (Doma and Parker, 2006), altering RNA stability by targeting the 5′ cap (Rand et al., 2005) or altering polyadenylation sites (Fig. 3B, v) (Vickers et al., 2001), targeting 5′ or 3′ untranslated region (UTR) regulatory elements, such as upstream open reading frames (Fig. 3B, vi) (Toulme, 2001; Baker et al., 1997), inhibition of translation by steric blockade of ribosomal binding or the initiation process (Fig. 3B, vii) (Janssen et al., 2013; Huang et al., 2018) and modulation of microRNA (miRNA) (Fig. 3B, viii) (Liang et al., 2017, 2016). The variety of mechanisms now available increases the genes and disorders that are amenable to ASO-mediated gene modulation and thus the number of patients that may benefit from ASO therapies.

### Applying ASOs to OGID syndromes

There are several avenues that could be explored for the development of ASOs to treat OGID syndromes, including those that are patient specific and/or disorder specific, by targeting particular variants or altering the causative gene, respectively. Given their overlapping mechanisms, it may also be possible to treat multiple OGID syndromes by modulating common pathways. The primary causes of SS, TBRS and MALNS are heterozygous loss-of-function variants of their respective causative genes, resulting in haploinsufficiency of each protein. As such, disorder-specific ASOs could be designed to upregulate expression of *NSD1*, *DNMT3A* or *NFIX*, perhaps through targeting of UTR regulatory elements, altering the formation of secondary structures or modulating regulation by miRNAs. Additionally, ASOs could be used to modulate common pathways, such as the H3K36 histone modification deposited by NSD1 and required by DNMT3A and NFIX, as discussed in this Review. There are several factors that impact the homeostasis of H3K36 modification (reviewed previously) (Sharda and Humphrey, 2022) that may act as potential targets for ASO development, such as SETD2/3/5, SET and MYND domain containing 2 (SMYD2), SETMAR or NSD2/3. ASOs could also be designed to modulate regulators of these targets to indirectly alter their activity, although systemic effects must be carefully considered.

The specificity and diverse mechanisms of action make ASOs an excellent therapeutic option. Furthermore, their current approval in the treatment of various nervous system pathologies, including the precedent set by the rapid progression of Milasen from development to the clinic in 10 months, make ASOs an attractive option for personalised and more generalised therapeutics. Although the causative genes of OGID syndromes including SS, TBRS and MALNS differ, their overlapping phenotype and molecular mechanisms present a potential avenue for future therapeutic development.

## Conclusions

Although there have been many advents in the space of neurodevelopmental disorders in the past decades, there is much that remains to be done. This rings even more true for OGID syndromes, as many were not aetiologically distinct entities until recent years. This Review serves as an insight into the underlying causes of three OGID syndromes; however, the strategies for symptom management, as well as suggestions for where the research landscape could go to help develop better treatments for these patients, apply to all OGID syndromes characterised to date. We have discussed how ASO therapeutics could be applied for treating OGID syndromes, with exciting avenues being developed in this space. The research landscape as it stands is providing hope for patients and families for treatment options once thought of as out of reach, with these possibilities hopefully eventuating soon.
